# Antimicrobial Susceptibility Profiles of *Erysipelothrix rhusiopathiae* and *Riemerella anatipestifer* Isolates from Clinical Cases of Waterfowl in Hungary Between 2022 and 2023

**DOI:** 10.3390/antibiotics14050478

**Published:** 2025-05-08

**Authors:** Ádám Kerek, Ábel Szabó, Ákos Jerzsele

**Affiliations:** 1 Department of Pharmacology and Toxicology, University of Veterinary Medicine, István utca 2, 1078 Budapest, Hungary; szabo.abel@student.univet.hu (Á.S.); jerzsele.akos@univet.hu (Á.J.); 2 National Laboratory of Infectious Animal Diseases, Antimicrobial Resistance, Veterinary Public Health and Food Chain Safety, University of Veterinary Medicine, István utca 2, 1078 Budapest, Hungary

**Keywords:** *Erysipelothrix rhusiopathiae*, *Riemerella anatipestifer*, antimicrobial resistance, AMR, minimum inhibitory concentration, MIC, waterfowl, geese, ducks, Hungary

## Abstract

**Background:***Riemerella anatipestifer* and *Erysipelothrix rhusiopathiae* remain clinically significant pathogens in the waterfowl industry, causing substantial economic losses and posing potential zoonotic risks. Antimicrobial resistance (AMR) continues to spread in the poultry sector, making regular surveillance of bacterial isolates essential. **Methods**: In this study, eight *R. anatipestifer* and eighteen *E. rhusiopathiae* strains were isolated from clinical cases in Hungarian waterfowl between 2022 and 2023. Minimum inhibitory concentration (MIC) values were determined for antibiotics of veterinary and public health significance. **Results**: For *R. anatipestifer*, high resistance rates were observed for spectinomycin, lincomycin, and tiamulin, while beta-lactam antibiotics (amoxicillin, ceftriaxone, and imipenem) demonstrated strong efficacy. Among the *E. rhusiopathiae* isolates, resistance to amoxicillin (89%) and enrofloxacin (61%) was notable, whereas ceftriaxone and doxycycline exhibited moderate antibacterial effects. **Conclusions**: Our findings underscore the importance of targeted antimicrobial use in the waterfowl industry. Beta-lactam antibiotics remain effective, whereas rising resistance to fluoroquinolones and aminoglycosides raise serious concerns. Routine AMR surveillance and the adoption of alternative strategies are crucial for controlling infections and maintaining flock health.

## 1. Introduction

Antimicrobial resistance (AMR) is one of the most pressing global health challenges, primarily driven by the unregulated and improper use of antibiotics [1]. The widespread and often unwarranted application of antimicrobials, particularly in livestock production [2], including intensive poultry farming, contributes significantly to the emergence and dissemination of resistant bacterial strains [3]. The rise in and spread of antibiotic-resistant pathogens pose serious threats to animal health, food safety, and public health, especially concerning zoonotic pathogens associated with food-producing animals [4].

The dynamic growth of the waterfowl industry has further exacerbated this issue. According to a 2020 report, over 6.44 billion ducks and 720 million geese were raised globally for food production in 2019 [5]. In Hungary, waterfowl production constitutes a significant sector within the poultry industry, with 628,000 geese and 4,214,000 ducks raised in 2023 alone [6]. The extensive use of antibiotics in large-scale farming plays a crucial role in the prevention and treatment of infectious diseases; however, their excessive or inappropriate application can accelerate the development of antimicrobial resistance [7,8,9].

One of the focus points of this study is *Riemerella anatipestifer*, as it is a Gram-negative, non-spore-forming bacterium that causes substantial economic losses in the waterfowl industry worldwide [10,11,12]. This pathogen is responsible for acute and chronic septicemia, polyserositis, and high mortality rates, leading to severe animal health and economic consequences [13,14,15]. The infection primarily affects young ducks and geese, often manifesting in severe clinical signs, including diarrhea, lethargy, respiratory distress, and poor feed conversion efficiency, which contribute to high treatment costs [16,17]. Horizontal transmission occurs through direct contact and water and feed contamination, while wild birds may act as potential vectors [18,19,20], emphasizing the critical importance of stringent biosecurity measures [21].

Antimicrobial resistance presents a significant challenge in the treatment of *R. anatipestifer* infections [17]. Although most isolated strains remain susceptible to amoxicillin, enrofloxacin, chloramphenicol, and ceftiofur, resistance to aminoglycosides and polymyxins exceeds 90% [22]. Additionally, increasing resistance to fluoroquinolones and broad-spectrum antibiotics [22] necessitates the exploration of alternative treatment options, including probiotics [23], plant extracts and essential oils [24,25,26,27,28], antimicrobial peptides [29], medium-chain fatty acids [30], and vaccine development [17].

The other focal point of this study is *Erysipelothrix rhusiopathiae*, as it is a Gram-positive, non-spore-forming, zoonotic bacterium that has also become problematic for the waterfowl industry. In recent years, outbreaks of erysipelas have been increasingly reported in chickens, hens, and geese [31,32]. This bacterium originates from soil, infected animals, and their byproducts [33] and can cause septicemia, pododermatitis, and pericardial hemorrhages in geese, often with high mortality rates [34,35]. In humans, *E. rhusiopathiae* infections can present with clinical manifestations ranging from localized cellulitis to severe endocarditis [34].

Antibiotic therapy, particularly penicillin, is considered as effective against *E. rhusiopathiae*. However, resistance data on isolates from the poultry sector remain scarce [33]. The use of inadequate treatment protocols may contribute to the emergence of resistant strains and their dissemination within flocks, as well as their zoonotic transmission. Therefore, the integration of antimicrobial susceptibility testing with pharmacokinetic studies is crucial for optimizing treatment strategies [36].

Given these challenges, this study aims to determine the antimicrobial susceptibility profiles of *R. anatipestifer* and *E. rhusiopathiae* strains isolated from clinical outbreaks in Hungary between 2022 and 2023, focusing on antimicrobials relevant to both animal and public health.

## 2. Results

### 2.1. Susceptibility of Riemerella anatipestifer Strains

During the one-year study, a total of eight *R. anatipestifer* strains associated with clinical outbreaks were identified from samples submitted to the national reference laboratory. The antimicrobial susceptibility profiles of these strains were analyzed, with particular emphasis on MIC_50_ and MIC_90_ values. The resistance patterns of the isolates exhibited substantial variation across different antimicrobial agents.

The distribution of MIC values for antibiotics with established clinical breakpoints is presented in Table 1, while Table 2 summarizes the MIC values for agents lacking defined breakpoints. The exact MIC values are provided in the Supplementary Materials.

**Figure 1:**
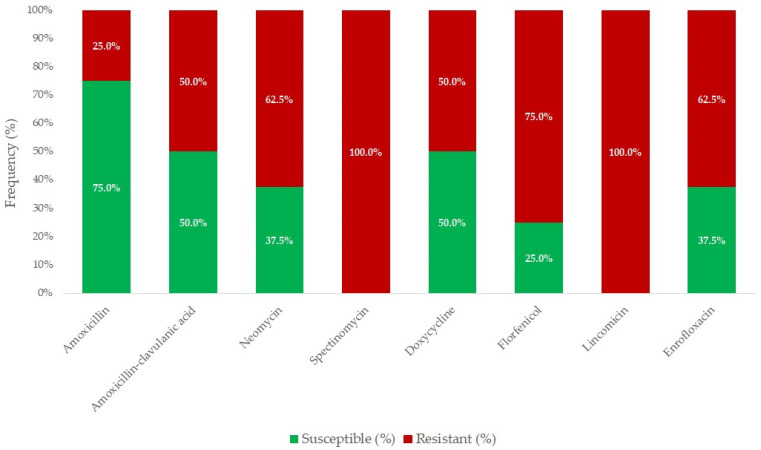
Antimicrobial resistance profile of *Riemerella anatipestifer* isolates (*n* = 8) for agents with established clinical breakpoints.

Figure 1 summarizes the susceptibility of *R. anatipestifer* strains to antimicrobials with established clinical breakpoints. The majority of the isolates (75.0%) were susceptible to amoxicillin; however, only half of the strains remained susceptible when tested against amoxicillin–clavulanic acid (MIC_90_ = 16 µg/mL and 64 µg/mL). In contrast, all the isolates were resistant to spectinomycin and lincomycin. Moderate resistance was detected for neomycin, doxycycline, florfenicol, and enrofloxacin (MIC_90_ = 32–64 µg/mL).

For antibiotics without defined clinical breakpoints, high levels of resistance were observed for lincomycin, spectinomycin, tiamulin, tilmicosin, and vancomycin. With MIC_90_ values ranging from 128 to 1024 µg/mL, these antimicrobials demonstrated limited therapeutic efficacy or complete inefficacy against *R. anatipestifer*. The lowest resistance levels were observed for colistin and imipenem, with MIC_90_ values of 0.125 and 1 µg/mL, respectively, suggesting that most of the isolates remain susceptible to these agents.

**Figure 2:**
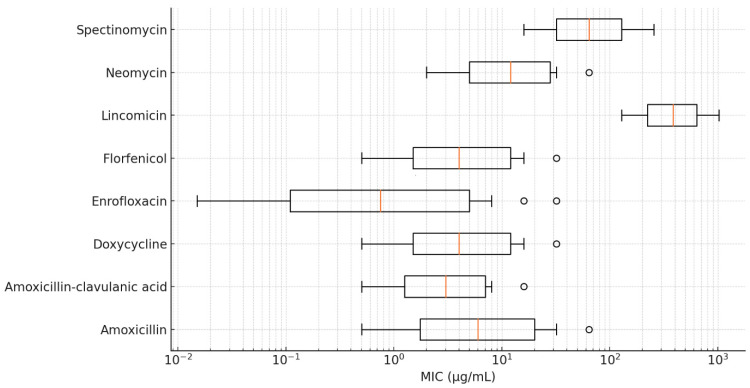
Distribution of minimum inhibitory concentration (MIC) values for *Riemerella anatipestifer* isolates with established clinical breakpoints, visualized using a box plot.

**Figure 3:**
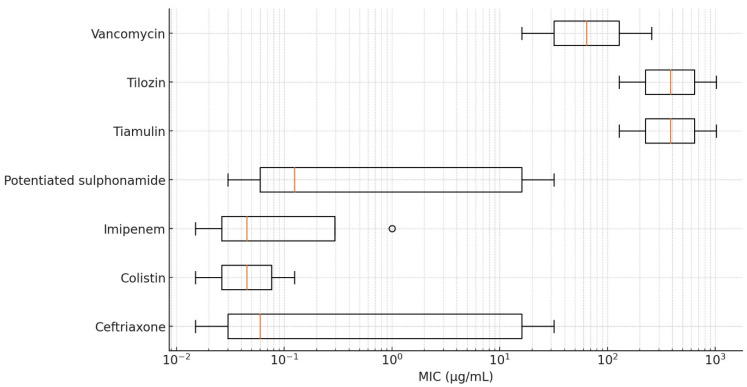
Distribution of minimum inhibitory concentration (MIC) values for *Riemerella anatipestifer* isolates without established clinical breakpoints, visualized using a box plot.

A logistic regression analysis revealed that geographical regions and sites of isolation were not significant predictors of resistance. However, because of the limited sample size, these results should be interpreted with caution. Cross-resistance analysis indicated the presence of partial resistance patterns between fluoroquinolones and tetracyclines, suggesting potential shared resistance mechanisms. The detailed MIC values are visualized using box plots presented in Figure 2 and Figure 3.

### 2.2. Susceptibility of Erysipelothrix rhusiopathiae Strains

**Figure 4:**
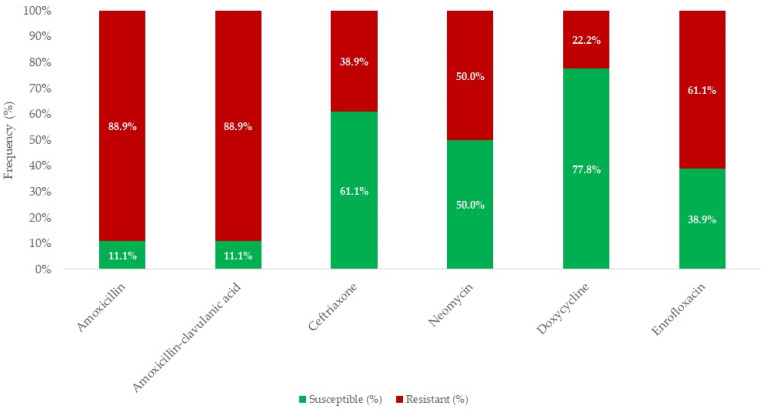
Antimicrobial resistance profile of *Erysipelothrix rhusiopathiae* isolates (*n* = 18) for agents with established clinical breakpoints.

**Figure 5:**
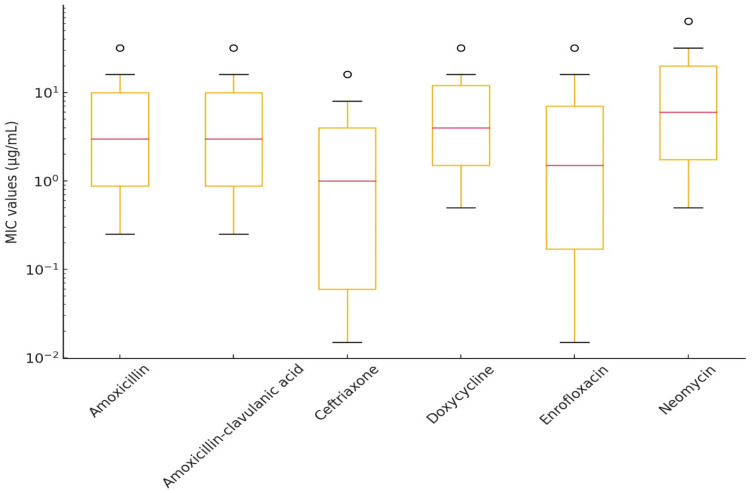
Distribution of minimum inhibitory concentration (MIC) values for *Erysipelothrix rhusiopathiae* isolates with established clinical breakpoints, visualized using a box plot.

**Figure 6:**
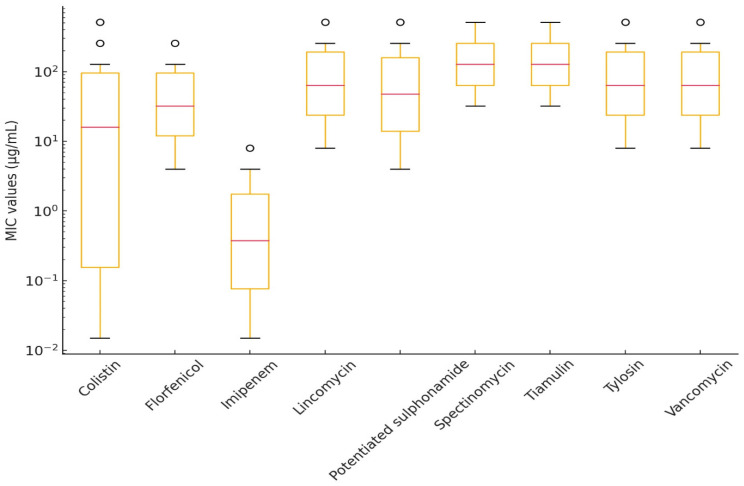
Distribution of minimum inhibitory concentration (MIC) values for *Erysipelothrix rhusiopathiae* isolates without established clinical breakpoints, visualized using a box plot.

A total of 18 *E. rhusiopathiae* strains isolated from clinical cases were tested for antimicrobial susceptibility, using MIC determination. Figure 4 summarizes the results of various antibiotics against *E. rhusiopathiae* isolates. The MIC distribution of antibiotics with established clinical breakpoints is presented in Table 3, while Table 4 contains the MIC values for agents without breakpoints. The detailed MIC values are visualized using box plots presented in Figure 5 and Figure 6.

Overall, only 11% of the strains were susceptible to amoxicillin and amoxicillin–clavulanic acid. In contrast, the highest susceptibility was observed for doxycycline (77.8%), followed by ceftriaxone (61.1%) and neomycin (50.0%).

Among beta-lactam antibiotics, ceftriaxone exhibited the highest efficacy (MIC_50_ = 0.5 µg/mL, MIC_90_ = 16 µg/mL), whereas amoxicillin and amoxicillin–clavulanic acid displayed higher MIC values (MIC_50_ = 4 and 2 µg/mL, MIC_90_ = 32 µg/mL), suggesting reduced susceptibility in a subset of *E. rhusiopathiae* isolates.

Among fluoroquinolones, enrofloxacin had an MIC_90_ of 32 µg/mL, which may indicate resistance development. Imipenem showed low MIC_50_ and MIC_90_ values (0.25 µg/mL and 2 µg/mL, respectively), though its clinical significance requires further investigation. Colistin and florfenicol exhibited broad MIC distributions; however, the MIC_90_ for colistin (64 µg/mL) suggests that some strains may naturally exhibit reduced susceptibility.

For macrolides and lincosamides (e.g., lincomycin and tylosin), high MIC_50_ and MIC_90_ values were observed (MIC_90_ ≥ 512 µg/mL), indicating that these antibiotics are likely ineffective against *E. rhusiopathiae* isolates. Tiamulin and spectinomycin also demonstrated reduced susceptibility (MIC_90_ = 512 µg/mL), raising the possibility of horizontally transferred resistance mechanisms.

**Figure 7:**
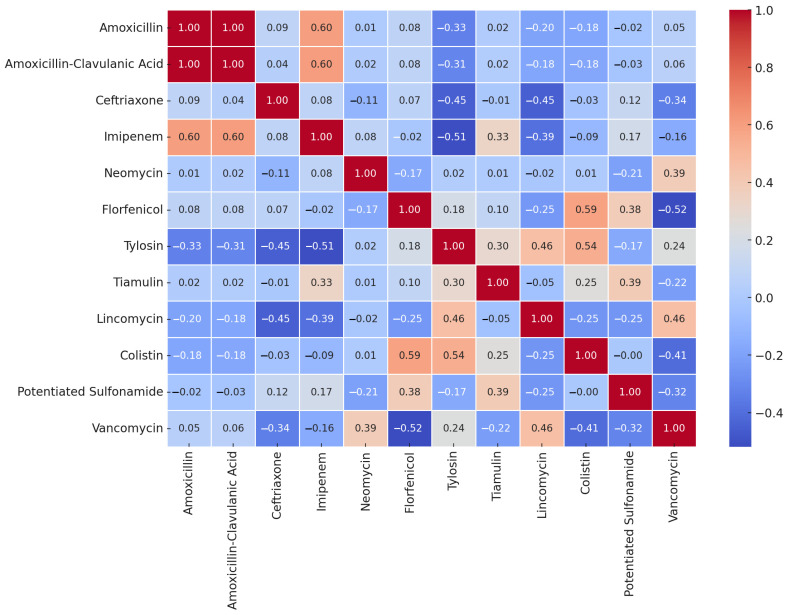
Correlation analysis of *Erysipelothrix rhusiopathiae* strains (*n* = 18) isolated from waterfowl for MIC values of different antibiotics.

The sample size of *E. rhusiopathiae* allowed us to perform a correlation analysis of the MIC values (Figure 7). Some antibiotics exhibited a strong positive correlation in their MIC values, which may suggest that resistance to these agents often occurs together. Examples include amoxicillin and amoxicillin–clavulanic acid (1.00), imipenem with amoxicillin (0.6) and amoxicillin–clavulanic acid (0.6), florfenicol and colistin (0.59), as well as tylosin and colistin (0.54). In contrast, we observed a negative correlation between florfenicol and vancomycin (−0.52), imipenem and tylosin (−0.51), ceftriaxone and tylosin (−0.45), and ceftriaxone and lincomycin (−0.45).

## 3. Discussion

In this study, we determined the antimicrobial susceptibility profiles of eight *R. anatipestifer* and eighteen *E. rhusiopathiae* clinical isolates against antimicrobial agents of veterinary and public health significance. The findings revealed substantial differences in susceptibility patterns among the isolates, highlighting the reduced efficacy of several conventionally used antibiotics.

Both *R. anatipestifer* and *E. rhusiopathiae* are typically associated with systemic infections in waterfowl, often presenting with macroscopic lesions, such as hepatomegaly, splenomegaly, fibrinous pericarditis, and meningoencephalitis. Histopathological examination may reveal multifocal necrosis, vascular damage, and infiltration by inflammatory cells in affected organs [37,38].

Few studies have evaluated antimicrobial susceptibility using MIC-based approaches, despite their greater reproducibility and efficiency compared to those of solid-medium-based methods. As a result, for both *R. anatipestifer* and *E. rhusiopathiae*, clinical breakpoints are not established in the literature for many antimicrobial agents. Therefore, our analysis focused on MIC_50_ and MIC_90_ values, which represent the antibiotic concentrations required to inhibit 50% and 90% of the tested bacterial population, respectively. In the absence of clinical breakpoints, these MIC values provide a useful reference for veterinarians in selecting appropriate antimicrobial therapies.

Our findings confirm that resistant strains of *R. anatipestifer* remain prevalent in waterfowl, representing a potential risk for poultry flocks and other livestock. In general, beta-lactam antibiotics, such as amoxicillin, amoxicillin–clavulanic acid, ceftriaxone, and imipenem, showed strong antimicrobial activities, with low MIC_50_ values but elevated MIC_90_ values, suggesting the presence of resistant subpopulations. Similarly, colistin retained good efficacy against *R. anatipestifer*, contrasting with certain previous studies that have reported higher resistance rates [39].

Although MIC_50_ values remained low (2, 0.5, 0.06, and 0.06 μg/mL, respectively), MIC_90_ values were elevated (64, 16, and 32 μg/mL), except for imipenem (1 μg/mL). Similar findings were reported by Shousha et al. in 2021, who also noted increased MIC values for cephalosporins [40]. However, our 75.0% susceptibility rate for amoxicillin was consistent with the results of Li et al. (2016) [41].

Interestingly, the observed lower susceptibility to amoxicillin–clavulanic acid compared to amoxicillin alone in *R. anatipestifer* strains may hypothetically be related to the presence of beta-lactamase enzymes with reduced sensitivity to clavulanic acid, such as AmpC-type variants. Although this mechanism is documented in certain Gram-negative bacteria, no molecular confirmation is currently available for *R. anatipestifer*. Therefore, this remains a putative explanation that requires further investigation. Given the lack of standardized susceptibility testing protocols and breakpoints for this species, minor variations in MIC values should also be interpreted cautiously [42]. Ceftriaxone susceptibility varied in previous studies: Hasan et al. (2022) reported 83.3% resistance [43], whereas Vo et al. (2022) observed only 5.8% resistance [14]. Despite their high efficacy rates, ceftriaxone and imipenem are not commonly used in poultry production.

Similarly, for enrofloxacin, the MIC_50_ value was low (2 μg/mL), but MIC_90_ was elevated (32 μg/mL), indicating the potential development of resistance. We identified a 62.5% resistance rate, compared to 99.3% reported by Zhu et al. [16] and 30% by Gyuris et al. [44]. These differences may stem from variations in sampling locations, bacterial strains, or local antibiotic usage patterns. The high MIC_90_ value further supports the notion that a subset of isolates exhibits markedly increased resistance, which may contribute to treatment challenges and the need for continuous surveillance.

In contrast, for neomycin, our findings support the existing literature indicating an increasing trend of acquired resistance to aminoglycosides [22]. We observed a 62.5% resistance rate, aligning with the 69.6% resistance reported by Chang et al. in 2019 [39].

For florfenicol, we detected 75.5% resistance, which is lower than the 97.9% reported by Gyuris et al. [44] but higher than the 8.6% found by Vo et al. [14]. Hasan et al. did not detect any resistant strains [43]. These discrepancies may be attributed to differences in geographical regions, bacterial strains, or sample sources or variations in antibiotic usage policies. The relatively high resistance rate observed in our study suggests the widespread presence of florfenicol resistance, which could impact treatment efficacy and necessitates further monitoring to assess potential emerging resistance trends.

For doxycycline, we observed 50.0% resistance, consistent with 86.7% reported by Zhong et al. [45] and 60% by Gyuris et al. [44], while Vo et al. found lower resistance rates [14]. These variations may reflect differences in antibiotic exposure, the geographical distribution of resistant strains, or methodological factors in susceptibility testing. The moderate resistance level observed in our study suggests that doxycycline may still retain partial efficacy, but the presence of resistant isolates warrants careful monitoring to prevent further resistance development.

For potentiated sulfonamides, our study found MIC_50_ = 16 μg/mL and MIC_90_ = 32 μg/mL, compared to 92.4% resistance by Gyuris et al. [44] and 34.8% by Vo et al. [14]. These discrepancies may stem from variations in bacterial populations, prior antimicrobial exposure, or regional differences in sulfonamide usage. The relatively high MIC_90_ value in our study indicates that a subset of the isolates exhibits elevated resistance levels, emphasizing the need for continuous surveillance and potential reconsideration of sulfonamide-based treatment strategies.

Although vancomycin and lincomycin were included in the susceptibility testing panels, it is important to note that these antibiotics primarily exhibit activity against Gram-positive bacteria and are not clinically relevant for the treatment of infections caused by Gram-negative organisms, such as *R. anatipestifer*. Their inclusion in the susceptibility profiles was because of the standardized testing protocols to provide comprehensive antimicrobial resistance data across all the tested antibiotics. However, the interpretation of their activity against Gram-negative strains should be approached cautiously, and such data are presented only to document baseline susceptibility patterns and to confirm known intrinsic resistance, particularly in underreported species, such as *R. anatipestifer*, rather than for clinical decision-making purposes [46,47].

Although valuable data were obtained regarding the antimicrobial susceptibility profiles of *R. anatipestifer* isolates, the relatively low sample size (*n* = 8) represents a limitation that should be acknowledged. The small number of isolates may reduce the statistical power to detect subtle resistance patterns, increase the variability in observed resistance rates, and limit the generalizability of the findings to larger waterfowl populations. Therefore, the results concerning *R. anatipestifer* should be interpreted with caution, and future studies with larger sample sizes are warranted to validate these preliminary observations.

*E. rhusiopathiae* is a well-known cause of erysipelas in birds, often manifesting as an acute, fulminant infection. The disease typically emerges suddenly, with initial sporadic mortality, followed by a progressive increase in deaths over subsequent days. Mortality rates range from <1% to 50% [31,48,49], and, thus, the economic impact for farmers can be severe. Despite this, data on *E. rhusiopathiae* strains affecting waterfowl remain scarce, making this study a valuable contribution to this field.

Recent studies have reported resistant isolates in poultry, yet penicillin remains the first-choice treatment for *E. rhusiopathiae* infections [50]. For *E. rhusiopathiae*, we found that amoxicillin resistance was alarmingly high, underscoring the growing issue of antibiotic resistance in this species. Hess et al. (2023) identified multidrug-resistant isolates from chickens and turkeys, which were found to be resistant to penicillin G and ampicillin [51]. In addition, *E. rhusiopathiae* exhibits intrinsic resistance to vancomycin [33]. Our study highlights the urgent need for further genetic and pharmacokinetic investigations to refine treatment strategies.

In this study, we observed 89% resistance to amoxicillin and amoxicillin–clavulanic acid for the strains of *E. rhusiopathiae* that we tested. Given that amoxicillin is among the most commonly used antimicrobials in poultry farming, this high resistance rate is concerning [52].

For ceftriaxone, we identified 38.9% resistance, whereas Hess et al. recently found no resistance to third-generation cephalosporins [51]. An Australian study on *E. rhusiopathiae* in humans reported MIC_90_ values of 0.03 μg/mL for penicillin and 0.125 μg/mL for ceftriaxone, suggesting that these antibiotics remain effective for human infections caused by *E. rhusiopathiae* [53].

Contrary to studies on fluoroquinolone susceptibility in *E. rhusiopathiae*, conducted in the 2000s [54,55], our findings support recent reports from 2023, which indicate increasing resistance [50,51]. We detected 61.1% resistance to enrofloxacin, which may be attributed to its widespread use in waterfowl farming and selection pressure leading to *gyrA* mutations affecting DNA supercoiling [56].

Tetracycline resistance varies across studies [55,57,58]. We identified 22.2% resistance to doxycycline, which is known to be mediated by efflux pumps, ribosomal protection, or enzymatic inactivation [57].

The correlation analysis conducted on *E. rhusiopathiae* suggests that certain closely related antibiotics, such as amoxicillin and amoxicillin–clavulanic acid, exhibit strong correlations. In some cases, a similar mode of action may indicate cross-resistance, as seen with macrolides and lincosamides. If a negative correlation is observed, it may suggest that the antibiotics act through different mechanisms, and resistance to one does not necessarily coincide with resistance to the other.

Overall, we can conclude that if two antibiotics strongly correlate, resistance to one is likely to indicate reduced efficacy of the other as well. Conversely, if there is little to no correlation, a strain resistant to one antibiotic may still be susceptible to another, which is crucial information for therapeutic decision making.

Furthermore, the presence of multiple strong positive correlations among different antibiotics may indicate the occurrence of multidrug-resistant (MDR) strains. This suggests that these bacteria may exhibit resistance to multiple agents simultaneously, posing significant clinical challenges.

### Conclusions

This study provides valuable insights into the antimicrobial resistance patterns of *R. anatipestifer* and *E. rhusiopathiae* strains in the Hungarian waterfowl industry. The findings highlight a decline in the efficacy of several conventionally used antibiotics, particularly fluoroquinolones and aminoglycosides, while amoxicillin remains a highly effective therapeutic option among beta-lactam antibiotics for *R. anatipestifer* infections. Thus, beta-lactam antibiotics (particularly amoxicillin) should remain the first-line therapeutic agents, as the use of fluoroquinolones may be compromised because of increasing resistance.

However, for *E. rhusiopathiae*, the high prevalence of amoxicillin-resistant strains presents a serious veterinary challenge. Therefore, there is a need for further research into vaccination strategies and alternative antimicrobial agents to combat infections caused by *E. rhusiopathiae*.

Our results emphasize the importance of targeted antimicrobial stewardship programs and continuous surveillance, particularly in poultry farming, where high antibiotic usage can drive resistance development. Implementing evidence-based antimicrobial stewardship strategies, such as optimizing treatment protocols, reducing unnecessary antibiotic use, and promoting alternative disease management approaches (e.g., vaccination and probiotics), can help to mitigate resistance emergence. Continuous surveillance is crucial for detecting shifts in resistance patterns, enabling timely interventions, and informing policy decisions to ensure the long-term efficacy of available antibiotics.

Future research should focus on the molecular characterization of resistance mechanisms and the role of horizontal gene transfer in these bacterial populations, as understanding the genetic basis of resistance is crucial for effective intervention strategies. Whole-genome sequencing and plasmid analysis could identify key resistance determinants, while transcriptomic studies may reveal regulatory pathways involved in antimicrobial resistance. Investigating horizontal gene transfer is particularly important, as mobile genetic elements can facilitate the spread of resistance genes within and across bacterial species, potentially compromising treatment efficacy. Such insights would support the development of targeted antimicrobial policies and alternative therapeutic approaches.

## 4. Materials and Methods

### 4.1. Origin of the Strains

Sample collection began in February 2022 and continued until May 2023. Ethical approval was not required, as no live animals were involved in the study. Necropsies and bacteriological isolations were performed by the National Food Chain Safety Office, Veterinary Diagnostic Directorate (Hungary), and the resulting isolates were provided for analysis. The isolates were derived from the liver and brain chamber and were cultured on blood agar (Biolab Zrt., Budapest, Hungary) under aerobic conditions at 37 °C for 48 h.

For *E. rhusiopathiae*, small, grayish-transparent colonies with alpha-hemolysis zones were observed. Gram staining confirmed that the slender bacterial cells belonged to the Gram-positive group. For *R. anatipestifer*, grayish-white, glistening, moist colonies were observed. The strains were incubated under 5% CO_2_, and Gram staining confirmed their classification as Gram-negative bacteria.

The isolates were identified using MALDI-TOF MS (Flextra-LAB Kft., Budapest, Hungary), with protein extraction performed using absolute ethanol, formic acid, and acetonitrile (Sigma-Aldrich, Steinheim, Germany) [59]. Mass spectrometry analysis was conducted using the MALDI Biotyper 12.0 software package (Bruker Daltonics GmbH, Bremen, Germany, 2024) [59].

The pure cultures obtained on Petri dishes were cryopreserved using the Microbank™ system (Pro-Lab Diagnostics, Richmond Hill, ON, Canada) at −80 °C until further use. Each sample was assigned a unique identifier, and information regarding the host species (duck or goose), organ of origin (brain chamber or liver), and collection site (geographical location) was recorded.

### 4.2. Preparation of Antimicrobial Stock Solutions

Stock solutions of the tested antimicrobial agents (Merck KGaA, Darmstadt, Germany) were prepared following the Clinical Laboratory Standards Institute (CLSI) guidelines [60]. Amoxicillin and amoxicillin–clavulanic acid (in a 2:1 ratio) were dissolved in phosphate buffer (pH 7.2, 0.01 mol/L). Imipenem was dissolved in phosphate buffer (pH 6.0, 0.1 mol/L). Ceftriaxone, doxycycline, spectinomycin, neomycin, colistin, tiamulin, tylosin, lincomycin, and vancomycin were dissolved in distilled water. For potentiated sulfonamides (sulfamethoxazole and trimethoprim, in a 20:1 ratio), sulfamethoxazole was dissolved in hot distilled water with a few drops of 2.5 mol/L NaOH, while trimethoprim was dissolved in 0.05 mol/L HCl in distilled water. Enrofloxacin was prepared using a few drops of 1 mol/L NaOH in distilled water. Florfenicol was dissolved using a few drops of 95% ethanol and distilled water.

All the stock solutions were prepared at an initial concentration of 1024 µg/mL for further dilution.

### 4.3. Determination of Minimum Inhibitory Concentrations

The phenotypic expression of AMR was assessed by determining minimum inhibitory concentration (MIC) values for each bacterial isolate. MIC testing was conducted following the CLSI guidelines [60], with breakpoints defined according to the CLSI, the European Committee on Antimicrobial Susceptibility Testing (EUCAST), and the relevant literature.

Bacterial strains stored at −80 °C were inoculated in 3 mL of Mueller–Hinton broth (MHB) and incubated at 37 °C for 18–24 h prior to testing. The MIC assays were performed using 96-well microtiter plates (VWR International, LLC, Debrecen, Hungary). All the wells except for those in the first column were filled with 90 µL of MHB. The initial stock solutions (512 µg/mL) were serially diluted in a twofold dilution series, covering a concentration range from 512 µg/mL to 0.0009 µg/mL. Then, 180 µL of the diluted stock solution was pipetted into the wells in the first column, followed by twofold serial dilutions across the plate. The excess solution was discarded after column 10, leaving 90 µL per well.

A bacterial suspension was adjusted to the 0.5 McFarland standard (5 × 10⁵ CFU/mL) using a nephelometer (ThermoFisher Scientific, Budapest, Hungary). Then, 10 µL of the standardized suspension was added per well, moving backward from column 11 [60]. Column 11 served as the positive control (containing only the bacteria and broth). Column 12 served as the negative control (containing only the broth).

The plates were incubated at 41 °C for 18–24 h, and MIC values were recorded using the Sensititre™ SWIN™ automatic MIC reader (ThermoFisher Scientific, Budapest, Hungary) and VIZION system software v. 3.4 (ThermoFisher Scientific, Budapest, Hungary, 2024). The reference strains used in the study were *R. anatipestifer* ATCC 11845 and *E. rhusiopathiae* ATCC 19414. The results were interpreted according to the CLSI standards [61,62], and MIC_50_ and MIC_90_ values were determined using population analysis [63].

### 4.4. Statistical Analyses

Statistical analyses were conducted using R software v. 4.1.0 [64]. The Shapiro–Wilk test was applied to assess the normality of the data. Non-parametric tests were performed when the data did not follow a normal distribution. The Kruskal–Wallis test was used to compare resistance levels across different antimicrobial agents. This test does not assume normality and is suitable for comparing median differences across multiple groups. Antimicrobial susceptibility classification (susceptible or resistant) was determined based on MIC values relative to clinical breakpoints.

Correlation analyses were conducted to identify associations between different antimicrobial agents. Pearson’s correlation coefficients were calculated to determine the strength and direction of relationships between the antibiotics’ MIC values. A correlation heatmap was generated to visualize significant associations, with positive correlations indicating a tendency for co-resistance and negative correlations suggesting independent resistance mechanisms. Statistical calculations and data visualization were conducted using Python v. 3.13.3. (and the pandas, seaborn, and matplotlib libraries).

For pairwise comparisons, a post hoc Mann–Whitney U test was applied. To reduce type I errors from multiple comparisons, a Bonferroni correction was used; however, this method increases the risk of type II errors (failing to detect true differences).

## Figures and Tables

**Table 1:** Frequency distribution of minimum inhibitory concentration (MIC) values for *Riemerella anatipestifer* isolates (*n* = 8) for antimicrobial agents with established clinical breakpoints. The vertical red lines indicate the clinical breakpoints.

Antibiotic(s)	Breakpoint (μg/mL)	Range (μg/mL)	Distribution of Strains by MIC (μg/mL)																	MIC_50_ (µg/mL)	MIC_90_ (µg/mL)
			0.015	0.03	0.06	0.125	0.25	0.5	1	2	4	8	16	32	64	128	256	512	1024		
Amoxicillin	64	0.015–1024						3	0	2	0	1	0	0	2					2	64
Amoxicillin–clavulanic acid ^1^	1	0.015–1024						4	0	0	1	1	1	0	1					0.5	16
Doxycycline	32	0.015–1024						1	1	0	2	0	0	3	1					4	32
Enrofloxacin	1	0.015–1024	1	2	0	0	0	0	0	1	0	0	2	2						2	32
Florfenicol	8	0.015–1024						1	0	0	1	1	3	1	0	1				16	32
Lincomycin	16	0.015–1024														3	2	1	2	256	1024
Neomycin	16	0.015–1024							1	2	0	0	2	0	3					16	64
Spectinomycin	4	0.015–1024													6	0	2			64	256

^1^ in a ratio of 2:1.

**Table 2:** Frequency distribution of minimum inhibitory concentration (MIC) values for *Riemerella anatipestifer* isolates (*n* = 8) for antimicrobial agents without established clinical breakpoints.

Antibiotic	Range (μg/mL)	Distribution of Strains by MIC (μg/mL)																	MIC_50_ (µg/mL)	MIC_90_ (µg/mL)
		0.015	0.03	0.06	0.125	0.25	0.5	1	2	4	8	16	32	64	128	256	512	1024		
Ceftriaxone	0.015–1024	1	2	2	0	0	0	0	0	0	0	2	1						0.06	32
Colistin	0.015–1024	2	2	0	0	0	0	0	0	0	0	0	3	0	0	1			0.03	0.125
Imipenem	0.015–1024	1	1	2	0	1	0	3											0.06	1
Potentiated sulfonamide ^1^	0.015–1024		1	0	1	0	0	0	0	0	0	3	2						16	32
Tiamulin	0.015–1024														3	3	0	2	256	1024
Tylosin	0.015–1024															5	1	2	256	1024
Vancomycin	0.015–1024											2	1	3	0	2			64	256

^1^ trimethoprim and sulfamethoxazole in a ratio of 19:1.

**Table 3:** Frequency distribution of minimum inhibitory concentration (MIC) values for *Erysipelothrix rhusiopathiae* isolates (*n* = 18) for antimicrobial agents with established clinical breakpoints. The vertical red lines indicate the clinical breakpoints.

Antibiotic(s)	Breakpoint (μg/mL)	Range (μg/mL)	Distribution of Strains by MIC (μg/mL)																	MIC_50_ (µg/mL)	MIC_90_ (µg/mL)
			0.015	0.03	0.06	0.125	0.25	0.5	1	2	4	8	16	32	64	128	256	512	1024		
Amoxicillin	0.5	0.015–1024				1	1	3	0	4	2	3	1	1	2					4	32
Amoxicillin–clavulanic acid ^1^	0.5	0.015–1024					2	3	1	4	2	2	1	1	2					2	32
Ceftriaxone	2	0.015–1024	3	3	2	0	0	2	1	0	2	2	1	1	0	1				0.5	16
Doxycycline	16	0.015–1024						3	2	4	4	1	2	1	1					2	16
Enrofloxacin	1	0.015–1024	1	2	2	0	0	2	2	0	1	2	2	2	0	1	1			1	32
Neomycin	32	0.015–1024						1	2	0	2	1	3	5	3	0	0	1		16	64

^1^ in a ratio of 2:1.

**Table 4:** Frequency distribution of minimum inhibitory concentration (MIC) values for *Erysipelothrix rhusiopathiae* isolates (*n* = 18) for antimicrobial agents without established clinical breakpoints.

Antibiotic	Range (μg/mL)	Distribution of Strains by MIC (μg/mL)																	MIC_50_ (µg/mL)	MIC_90_ (µg/mL)
		0.015	0.03	0.06	0.125	0.25	0.5	1	2	4	8	16	32	64	128	256	512	1024		
Colistin	0.015–1024	3	1	1	0	4	0	0	1	0	0	1	4	1	0	1	1		0.25	64
Florfenicol	0.015–1024								3	3	5	0	1	1	1	4			8	256
Imipenem	0.015–1024	2	1	1	4	3	2	2	1	2									0.25	2
Lincomycin	0.015–1024								1	0	1	0	0	0	2	2	6	6	512	1024
Potentiated sulfonamide ^1^	0.015–1024						1	0	3	3	1	0	0	1	1	1	3	4	64	1024
Spectinomycin	0.015–1024												4	7	2	2	1		64	512
Tiamulin	0.015–1024													2	4	9	3		256	512
Tylosin	0.015–1024						1	1	0	0	1					2	10	3	512	1024
Vancomycin	0.015–1024						3	1	0	1	0	1	0	0	0	8	4		256	512

^1^ trimethoprim and sulfamethoxazole in a ratio of 19:1.

## Data Availability

The data presented in this study are available from the corresponding author upon reasonable request.

## Supplementary Materials

The following supporting information can be downloaded at https://www.mdpi.com/article/10.3390/antibiotics14050478/s1: Supplementary Materials excel file.

## Abbreviations

The following abbreviations are used in this manuscript:

AMR	Antimicrobial resistance
CLSI	Clinical Laboratory Standards Institute
CFU	Colony-forming unit
EUCAST	European Committee on Antimicrobial Susceptibility Testing
MHB	Mueller–Hinton broth
MIC	Minimum inhibitory concentration
*R. anatipestifer*	*Riemerella anatipestifer*
*E. rhusiopathiae*	*Erysipelothrix rhusiopathiae*
